# Vimentin is a key regulator of cell mechanosensing through opposite actions on actomyosin and microtubule networks

**DOI:** 10.1038/s42003-024-06366-4

**Published:** 2024-05-29

**Authors:** Farid Alisafaei, Kalpana Mandal, Renita Saldanha, Maxx Swoger, Haiqian Yang, Xuechen Shi, Ming Guo, Heidi Hehnly, Carlos A. Castañeda, Paul A. Janmey, Alison E. Patteson, Vivek B. Shenoy

**Affiliations:** 1 Center for Engineering Mechanobiology, University of Pennsylvania, Philadelphia, PA 19104 USA; 2 Department of Mechanical and Industrial Engineering, New Jersey Institute of Technology, Newark, NJ 07102 USA; 3 Institute for Medicine and Engineering, University of Pennsylvania, 3340 Smith Walk, Philadelphia, PA 19104 USA; 4 Physics Department, Syracuse University, Syracuse, NY 13244 USA; 5 BioInspired Institute, Syracuse University, Syracuse, NY 13244 USA; 6 Department of Mechanical Engineering, Massachusetts Institute of Technology, Cambridge, MA 02139 USA; 7 Department of Biology, Syracuse University, Syracuse, NY 13244 USA; 8 Departments of Biology and Chemistry, Syracuse University, Syracuse, NY 13244 USA; 9 Interdisciplinary Neuroscience Program, Syracuse University, Syracuse, NY 13244 USA; 10 Departments of Physiology, and Physics & Astronomy, University of Pennsylvania, Philadelphia, PA 19104 USA; 11 Department of Materials Science and Engineering, School of Engineering and Applied Science, University of Pennsylvania, Philadelphia, PA 19104 USA

**Keywords:** Biophysics, Computational biophysics

## Abstract

The cytoskeleton is a complex network of interconnected biopolymers consisting of actin filaments, microtubules, and intermediate filaments. These biopolymers work in concert to transmit cell-generated forces to the extracellular matrix required for cell motility, wound healing, and tissue maintenance. While we know cell-generated forces are driven by actomyosin contractility and balanced by microtubule network resistance, the effect of intermediate filaments on cellular forces is unclear. Using a combination of theoretical modeling and experiments, we show that vimentin intermediate filaments tune cell stress by assisting in both actomyosin-based force transmission and reinforcement of microtubule networks under compression. We show that the competition between these two opposing effects of vimentin is regulated by the microenvironment stiffness. These results reconcile seemingly contradictory results in the literature and provide a unified description of vimentin’s effects on the transmission of cell contractile forces to the extracellular matrix.

## Abstract (web summary)

Vimentin tunes cell stress by assisting in both actomyosin force transmission and reinforcement of microtubule networks under compression. The competition between these two opposing effects of vimentin is regulated by the microenvironment stiffness.

## Introduction

Cells in the body experience different physical stresses in the form of tension, compression, and shear during physical activities, such as muscle contraction, breathing, blood flow, body movement, and sleep^1^. It is now well known that these forces play important roles in many biological processes including tissue and organ morphogenesis, proliferation, differentiation, and gene expression^1,2^. More than a century ago, it was proposed that in addition to these “external” forces, non-muscle cells generate “internal” forces and transmit these forces to their surrounding extracellular matrices (ECMs) through focal adhesions^3^. It was later shown that these internally generated contractile forces enable adherent cells to sense and respond to the mechanical properties of their ECM. Cells generate higher traction forces when they are exposed to stiffer environments^4–6^ and engage in a mechanical cross-talk with their ECMs as these forces reorganize and alter the local mechanical properties of the ECMs^6,7^.

In non-muscle cells, the internal forces are generated by the actomyosin machinery. The myosin head domains bind to actin filaments and pull on them to generate internal contractile forces, which are transmitted to the ECM. Inhibition of either myosin motors or actin polymerization decreases cellular traction forces^8^. Unlike actin filaments, disruption of microtubules leads to an increase in cellular contractility^9^ and cell traction forces^10^, which has been attributed to the mechanical resistance of microtubules against actomyosin contractility^11,12^, and activation of GEF-H1 and the Rho-Rock pathway upon microtubule depolymerization^9,13^. Taken together, cell stress depends on the balance between actin filaments, which are under tension, and microtubules which mainly undergo compressive deformations^11,12,14^.

While the roles of the actin and microtubule networks in the generation and transmission of internal forces are known, it is not clear how intermediate filaments impact cellular forces. Vimentin is a type of intermediate filament expressed in mesenchymal cells and highly invasive cancer cells. Vimentin filaments are known to exhibit strain-stiffening behavior under physical loading^15–18^, and mechanically interact with microtubules and actomyosin filaments through cross-linkers such as plectin^19^. Emerging studies are revealing intricate (and sometimes contradictory) ways in which vimentin impacts cellular stress in fibroblasts, the main cell type in connective tissues. For example, vimentin has been found to either increase^20,21^ or decrease cell-generated stress^22–24^. In other cases, vimentin had little to no effect at all^25^. This discrepancy calls into question the role of intermediate filaments in the transmission of internally generated contractile forces to the ECM.

To answer this question, we introduce an active chemo-mechanical model that accounts for all three cytoskeletal polymer networks involved in the generation and transmission of cellular forces to the ECM. Combining modeling approaches with experimental data, we show that vimentin intermediate filaments (VIFs) can have two opposite effects on cellular forces depending on matrix stiffness, reconciling seemingly opposite results in the literature. Matrix stiffness is known to change with aging or conditions such as fibrosis, cancer progression, diabetes, and obesity^26,27^, and our results show that these changes in the stiffness of the extracellular environment affect the role of vimentin in cellular mechanosensing and crosstalk amongst all three cytoskeletal networks.

## Results

### Modeling the chemo-mechanical crosstalk between vimentin intermediate filaments, microtubules, and actin filaments

**Fig. 1:**
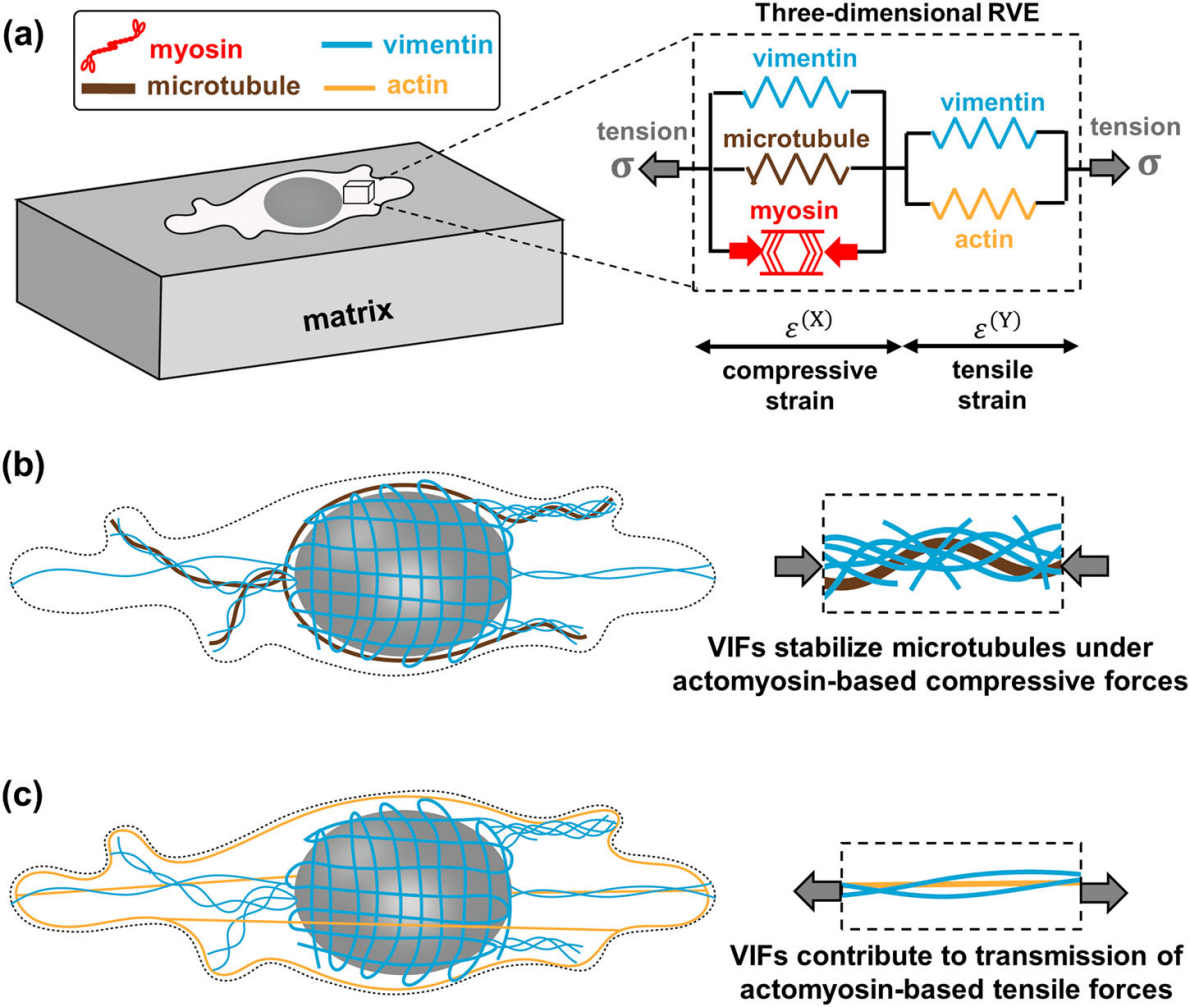
Mechanical crosstalk between cytoskeletal components. **a** The cell model includes (i) an active force-generating contractile element representing myosin motors, (ii) the actin filament network, (iii) the microtubule network, and (iv) two elements of the vimentin intermediate filament network. **b** The first vimentin element laterally reinforces and stabilizes microtubules under contractility-based compressive forces. **c** The second vimentin element interacts with actin filaments and is involved in the transmission of contractility-based tensile forces to the matrix.

The cytoskeleton is composed of three interconnected biopolymers: actin filaments, microtubules, and intermediate filaments. There is growing evidence that these three biopolymeric networks interact with each other and this interaction plays an important role in many cellular functions including cell migration and polarization^28^. To understand the role of intermediate filaments in the transmission of cellular forces to the ECM, we develop a chemo-mechanical model that accounts for some key cellular components involved in the generation and transmission of forces (Supplementary Figs. 1, 2). In this model, the cytoskeleton is comprised of (i) the myosin molecular motors, (ii) the microtubule network, (iii) the actin filament network, and (iv) the vimentin intermediate filament network (see Fig. 1a and Methods). In what follows, we first describe how actin, myosin, and microtubules in our model respond to ECM mechanics. We then add VIFs to the model and describe how the model predicts the existence of two families of VIFs with distinct functions required to consistently explain our experimental results.

#### Modeling the active cellular contraction

The first component of the model is myosin motors which generate internal contractility (Fig. 1a and Supplementary Fig. 1)^29^. We treat the average density of phosphorylated myosin motors as a symmetric tensor, ${{{{{{\rm{\rho }}}}}}}_{{ij}}$, (as required by conditions of moment balance) whose components represent cell contractility in different directions (see Supplementary Note 1.1)^30^. The cell contractility ${{{{{{\rm{\rho }}}}}}}_{{ij}}$ generates compressive stress ${{{\mbox{C}}}}_{{ijkl}}^{\left({{{{{\rm{X}}}}}}\right)}{{{{{{\rm{\varepsilon }}}}}}}_{{kl}}^{\left({{{{{\rm{X}}}}}}\right)}$ and tensile stress ${{{{{{\rm{\sigma }}}}}}}_{{ij}}$ in the cytoskeletal components that are in compression (e.g., microtubules) and tension (e.g., actin elements), respectively,

1 $${{{{{{\rm{\rho }}}}}}}_{{ij}}=-{{{\mbox{C}}}}_{{ijkl}}^{\left({{{{{\rm{X}}}}}}\right)}{{{{{{\rm{\varepsilon }}}}}}}_{{kl}}^{\left({{{{{\rm{X}}}}}}\right)}+{{{{{{\rm{\sigma }}}}}}}_{{ij}}$$

where ${{{\mbox{C}}}}_{{ijkl}}^{\left({{{{{\rm{X}}}}}}\right)}$ and ${{{{{{\rm{\varepsilon }}}}}}}_{{kl}}^{\left({{{{{\rm{X}}}}}}\right)}$ are the stiffness and strain tensors of the cytoskeletal components that are in compression (Fig. 1a).

#### Modeling the regulation of myosin phosphorylation level and force generation in response to mechanical signals

As tension is generated in the cytoskeleton (either by the intrinsic cell contractility or external tensile forces), cells increase their contractility through phosphorylation of more myosin motors which in turn generates higher cytoskeletal tension. This reciprocal mechanism is controlled by tension-activated signaling pathways such as the Rho-Rock and the Ca^2+^ pathways (Supplementary Fig. 1)^31,32^. To include the signaling pathways and capture this feedback mechanism, we assume that the average of contractility in all three directions, $\frac{1}{3}{\rho }_{{kk}}=({{{{{{\rm{\rho }}}}}}}_{11}+{{{{{{\rm{\rho }}}}}}}_{22}+{{{{{{\rm{\rho }}}}}}}_{33})/3$, increases with the average of tension in the cytoskeleton, $\frac{1}{3}{\sigma }_{{kk}}=({{{{{{\rm{\sigma }}}}}}}_{11}+{{{{{{\rm{\sigma }}}}}}}_{22}+{{{{{{\rm{\sigma }}}}}}}_{33})/3$, in the following form

2 $$\frac{{\rho }_{{kk}}}{3}={f}_{{{{{{\rm{m}}}}}}}\frac{{\sigma }_{{kk}}}{3}+{f}_{0}\,{\rho }_{0}\,$$

where this stress-dependent feedback mechanism is regulated by the feedback parameter ${f}_{{{{{{\rm{m}}}}}}}$. In the absence of tension (${\sigma }_{{kk}}=0$), ${f}_{0}$ regulates the mean contractility $\frac{1}{3}{\rho }_{{kk}}$ with ${\rho }_{0}$ being the basal cell contractility (see Supplementary Note 1.1 for ${f}_{{{{{{\rm{m}}}}}}}$ and ${f}_{0}$). We have shown that this positive feedback between contractility and cytoskeletal tension (Supplementary Fig. 2) consistently explains cell responses to mechanical signals such as matrix stiffness, cell spreading area, and external forces^33^. For example, as a result of the feedback mechanism, our simulations show that the cell contractility $\rho$ and the cell-generated tensile stress $\sigma$ increase with matrix stiffness and then reach a plateau (Supplementary Fig. 3), consistent with experimental observations^34^.

#### Modeling the polymerization of the actin network in response to tension

The second component of the model is actin filaments which are connected to the myosin element in series (Fig. 1a). Subsequently, the actin element experiences tension and transmits myosin-generated tensile forces to the extracellular matrix through focal adhesions as observed experimentally^35^. Starting with a uniform and isotropic distribution, the stiffness of the actin network ${{{\mbox{C}}}}_{{ijkl}}^{({{{{{\rm{A}}}}}})}$ increases in proportion and in the directions of the tensile principal components of the stress tensor ${{{{{{\rm{\sigma }}}}}}}_{{ij}}$,

3 $${{{\mbox{C}}}}_{{ijkl}}^{({{{{{\rm{A}}}}}})}={{{\mbox{C}}}}_{{ijkl}}^{({{{{{\rm{I}}}}}})}+{{{\mbox{C}}}}_{{ijkl}}^{({{{{{\rm{F}}}}}})}\,$$

where ${{{{{{\bf{C}}}}}}}^{({{{{{\rm{I}}}}}})}$ and ${{{{{{\bf{C}}}}}}}^{({{{{{\rm{F}}}}}})}$ are the initial and stiffening parts of the stiffness tensor ${{{{{{\bf{C}}}}}}}^{({{{{{\rm{A}}}}}})}$, respectively. Note that ${{{{{{\bf{C}}}}}}}^{({{{{{\rm{F}}}}}})}\, \approx \, 0$ for low tensile stress ${{{{{{\rm{\sigma }}}}}}}_{{ij}}$ and increases with tension (but not in compression), representing the formation of actin filaments and stress fibers in response to tension as observed experimentally (Supplementary Fig. 4)^36^. As a result of this stiffening and concomitant with phosphorylation of more myosin motors (Supplementary Fig. 5), the model shows that the cytoskeletal stiffness $C$ increases with matrix stiffness and then reaches a plateau (Supplementary Fig. 6) in agreement with our previous experiments (see Supplementary Notes 1.1 and 1.2)^37^. Furthermore, disruption of actin filaments in the model, by reducing the stiffness of the actin element ${C}^{({{{{{\rm{A}}}}}})}$, leads to decreases in cell contractility, cell-generated traction force, and cell-induced matrix deformation, which are all consistent with experimental measurements^38^ (Supplementary Fig. 7).

#### Modeling the increase in cell contractility in response to microtubule depolymerization

The third component of the model is microtubules, which are placed in parallel with the contractile element (Fig. 1a). As a result, microtubules in the model experience compression which is consistent with experimental observations^11,12^. Depolymerization of microtubules reduces their mechanical resistance against cell contraction and activates GEF-H1 and the Rho-Rock pathway in the model as demonstrated in experiments^9,11–13^ (Supplementary Fig. 8, see Supplementary Note 1.1). As a result, simulations of microtubule depolymerization, by reducing the stiffness of the microtubule element, show that cells become more contractile, and subsequently generate higher traction forces and stretch the matrix more (Supplementary Fig. 7), which all agree with experimental measurements^9,10,39^.

#### Addition of vimentin intermediate filaments to the model

We next study how intermediate filaments should be connected to other cytoskeletal components in the model. To this end, we focus on fibroblasts which are strongly contractile cells, and we study the effect of vimentin intermediate filaments on their contractile forces. Vimentin filaments, directly and indirectly, interact with both microtubule and actomyosin networks^19,28,40–43^. We, therefore, include two vimentin elements in the model, one in parallel with the microtubule element and the other in parallel with the actin element, to account for the interaction of vimentin with both microtubule and actomyosin networks (Fig. 1a–c). Using the model, we first study the effect of each of these vimentin elements, and their overall effect on cellular forces. We then experimentally validate the model predictions for fibroblasts cultured on different matrix stiffness.

#### Modeling actin-vimentin interactions

**Fig. 2:**
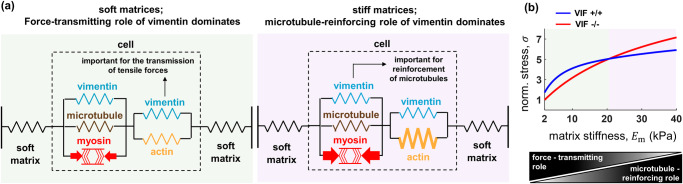
Disruption of the vimentin network can decrease or increase cell contractility depending on microenvironment stiffness (model predictions). **a** On the one hand, vimentin intermediate filaments (VIF) are involved in the transmission of actomyosin-based tensile forces to the matrix and therefore disruption of vimentin can decrease cell contractility and cell traction stress. On the other hand, vimentin filaments reinforce microtubules under compression and resist cell contraction. As a result, disruption of vimentin can lead to destabilization of microtubules and reduce the resistance against cell contraction, which can in turn increase cell traction stress. Cells on soft substrates are characterized by a weak actomyosin network and vimentin plays an important role in the transmission of forces to the substrate (force-transmitting role of VIF), while on stiff substrates, microtubules experience high compression, and they require reinforcement from the vimentin network to withstand the compression and resist cell contraction (microtubule-reinforcing role of VIF). **b** The model shows how the two opposing effects of vimentin compete in a matrix stiffness-dependent manner. For low matrix stiffness (low cellular contractility), the force-transmitting role of vimentin overpowers its microtubule-reinforcing role and therefore disruption of vimentin decreases cell contractile force. In contrast, for high matrix stiffness (high cellular contractility), disruption of vimentin increases cell contractile force as the microtubule-reinforcing role of vimentin becomes more important with increasing matrix stiffness.

In addition to non-physical interactions through biochemical signaling, intermediate filaments also interact with actin filaments through physical contact mediated by cross-linkers, direct binding, and steric effects^28^ as we have shown in our in vitro studies^40^. A large portion of VIFs that interact with contractile actin filaments are expected to experience tensile stresses (Fig. 1c)^44^. Therefore, the first vimentin element is placed in parallel with the actin element, and subsequently undergoes tensile stresses (Fig. 1a). This element represents the actomyosin-associated VIFs and promotes the transmission of contractility-based tensile forces to the ECM (Fig. 2a, top panel). We find that disruption of this vimentin element leads to decreases in the cell contractility $\rho$, the cell-generated stress $\sigma$, and the matrix strain ${\varepsilon }_{{{{{{\rm{m}}}}}}}$ (Supplementary Fig. 9).

#### Modeling microtubule-vimentin interactions

Microtubules interpenetrate a dense network of intermediate filaments which laterally reinforces and stabilizes microtubules under contractility-based compressive stresses (Fig. 1b)^14,45^. Thus, the second vimentin element is placed in parallel with the microtubule element (Fig. 1a), supporting microtubules under contractility-based compressive stresses and mitigating their destabilization (Fig. 2a, bottom panel). This negatively affects cellular forces as the high stiffness of the vimentin element prevents the contraction of the myosin element, thereby hindering the generation of contractile forces. Our simulations show that disruption of this vimentin element, by decreasing its stiffness in our model, increases the cell contractility $\rho$, the cell-generated stress $\sigma$, and the matrix strain ${\varepsilon }_{{{{{{\rm{m}}}}}}}$ (Supplementary Fig. 9).

Taken together, our model predicts that VIFs can exhibit opposite effects on cell contractility. On the one hand, disruption of vimentin decreases traction force due to the force-transmitting role of VIFs, while on the other hand, disruption of vimentin increases traction force due to the microtubule-reinforcing role of VIFs (Fig. 2a). We next study how these two opposite effects of vimentin compete with each other, and we examine whether this competition depends on matrix stiffness.

### The model predicts that vimentin filaments can increase or decrease active cellular forces depending on matrix stiffness

With the two VIF elements in the model, we simulate the disruption of vimentin filaments to study how lack of VIFs affects cellular forces at different matrix stiffness (Fig. 2b). Loss of vimentin is modeled by setting the stiffness of both VIF elements equal to zero. The model predicts that at low matrix stiffness (or when actomyosin contractility is low) the force-transmitting role of vimentin filaments is more prominent compared with their microtubule-reinforcing role, and therefore disruption of vimentin decreases the cell contractility $\rho$, the cell-generated stress $\sigma$, and the matrix strain ${\varepsilon }_{{{{{{\rm{m}}}}}}}$ (Fig. 2b and Supplementary Fig. 10). In contrast, the model shows that for cells on stiff substrates (cells with high levels of actomyosin contractility), the microtubule-reinforcing role of vimentin filaments dominates over their force-transmitting role, and therefore disruption of vimentin increases $\rho$, $\sigma$, and ${\varepsilon }_{{{{{{\rm{m}}}}}}}$ (Fig. 2b and Supplementary Fig. 10).

The results demonstrate that vimentin’s effect on cellular stress is regulated by matrix stiffness. Vimentin filaments are simultaneously involved in both the transmission of tensile forces to the matrix (force-transmitting role) and the reinforcement of microtubules under compression (microtubule-reinforcing role). In cells on soft substrates, actin filaments form a weak network and microtubules experience low compression. Therefore, the force-transmitting role of vimentin is more important than its microtubule-reinforcing role on soft substrates, and as a result, depletion of VIFs in cells on soft substrates decreases cellular forces. In contrast, in cells on stiff matrices, actin filaments form a strong contractile network that transmits tensile forces to the matrix, and microtubules experience high compression. Under these conditions, the microtubule-reinforcing role of vimentin is stronger than its actin-based force-transmitting effects. As a result, the model predicts depletion of VIFs in cells on stiff substrates causes more buckling and instability of microtubules and thus increases cellular forces.

### Experimental validation of model predictions: vimentin impacts cellular force and cell area in a matrix-dependent manner

**Fig. 3:**
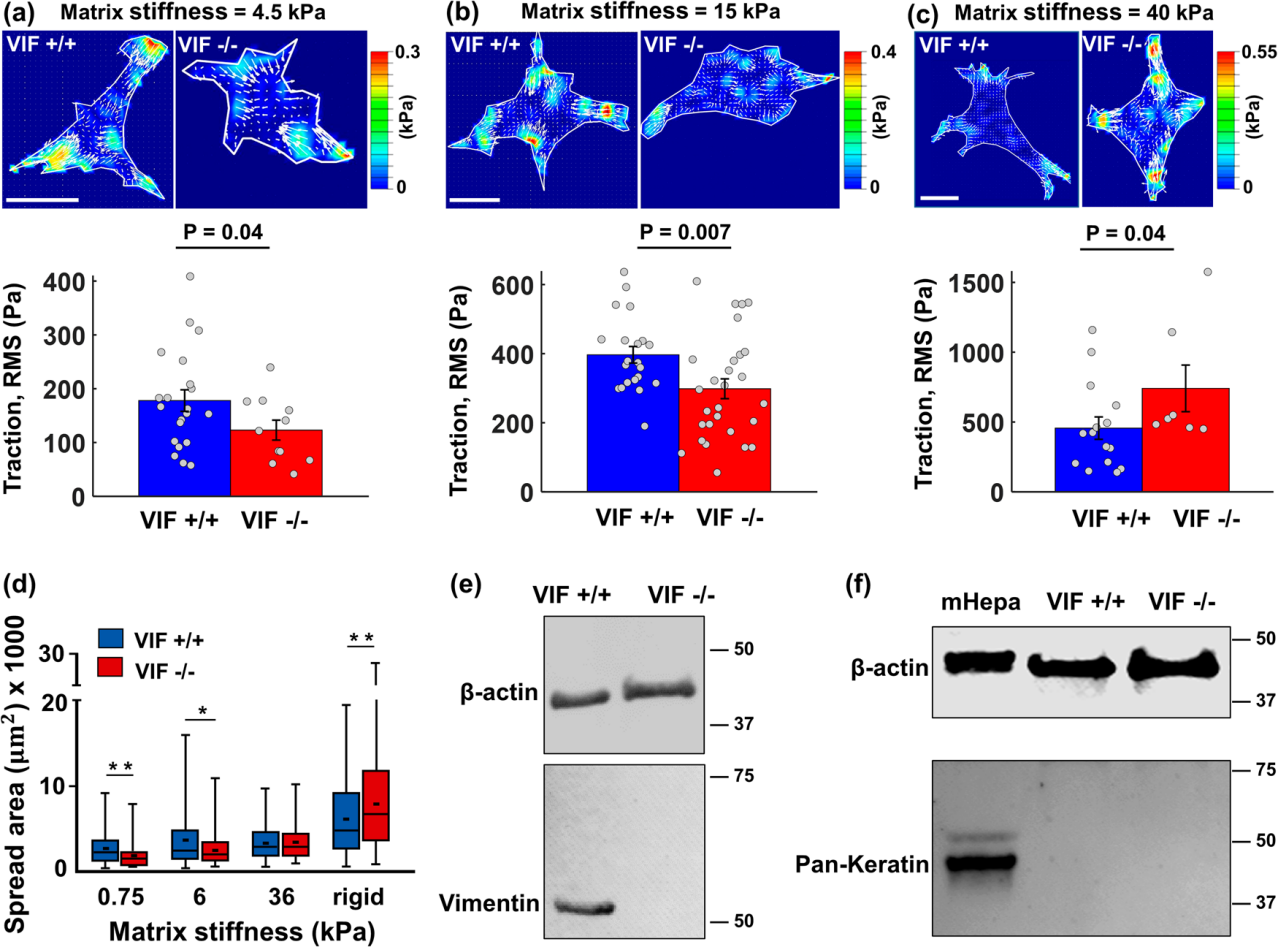
Matrix-stiffness dependent effect of vimentin on traction forces and spreading area (experimental validations). **a**–**c** VIF −/− cells generate lower traction forces on soft substrates (4.5 kPa elastic modulus with $n$ = 21 and 11, and 15 kPa elastic modulus with $n$ = 21 and 28), while they generate higher forces on stiff substrates (40 kPa elastic modulus with $n$ = 15 and 7). The unpaired Student’s t-test was used. The height of the bars and the error bars indicate the mean and the standard error, respectively. Traction stresses are the root mean square (RMS) values of cellular contractile forces per unit area. Scale bar: 20 µm. **d** Similarly, compared with wild-type cells, VIF −/− cells spread less on soft substrates, whereas they spread more on rigid substrates (all $n$ > 100). Panel (**d**) was reproduced from our previously published data in ref. ^46^. These results are consistent with the model predictions and confirm that vimentin is a key modulator of cell responses to ECM mechanics. **e** Western blot for vimentin from wild-type (VIF +/+) and vimentin-null (VIF −/−) mouse embryonic fibroblast cells. No vimentin was detected in VIF −/− cells. β-actin was used as a loading control. **f** Western blot for keratin from hepatocyte cells, wild-type (VIF +/+), and vimentin-null (VIF −/−) mouse embryonic fibroblast cells. No keratin was detected in either mouse embryonic fibroblast cell. Keratin from hepatocytes was used as a positive control, and β-actin was used as a loading control.

As illustrated in Fig. 2b, the model predicts that disruption of vimentin has opposite effects on the cellular force at different matrix stiffness, resulting in a crossover in VIF +/+ and VIF −/− curves. The model also shows that the matrix stiffness at which the crossover occurs depends on the physical properties of the vimentin filament network (Supplementary Figs. 11, 12). Therefore, to validate the model prediction and to examine whether the crossover occurs at a physically measurable and physiologically relevant matrix stiffness range, we culture wild-type (VIF +/+) and vimentin-null (VIF −/−) mouse embryonic fibroblast (mEF) cells on fibronectin-coated hydrogel substrates with different stiffness. Our western blot analysis confirms the depletion of vimentin filaments in VIF −/− mEF cells (Fig. 3e). Also, we note no keratin was detected in VIF +/+ and VIF −/− mEF cells (Fig. 3f).

We first perform traction force microscopy (TFM) experiments to directly measure contractile forces generated by VIF +/+ and VIF −/− cells on gels of varying stiffness (Methods). Figure 3 shows TFM results for cells on fibronectin-coated polyacrylamide gel with elastic moduli of 4.5, 15, and 30 kPa. While the average traction stress increases with substrate stiffness for each cell type, the impact of disrupting vimentin is different between the soft (4.5–15 kPa) and stiff substrates (40 kPa). The data show while cell traction stress generated by VIF −/− cells is higher than that of VIF +/+ cells on the stiff substrate (Fig. 3c and Supplementary Figs. 13–15), the opposite occurs on the soft substrates (Fig. 3a, b). While both VIF +/+ and VIF −/− cells sense and respond to matrix stiffening by increasing their traction forces, the increase of contractile forces in VIF −/− cells is more pronounced, making them more sensitive to matrix stiffening.

These results are consistent with our previous studies that vimentin impacts cell spreading area in a matrix stiffness-dependent manner^46^. Compared with cells containing vimentin, VIF −/− cells spread less on soft substrates (0.75 and 6 kPa), whereas they spread more on rigid substrates (Fig. 3d; original data from ref. ^46^). Since in many cell types spreading area is positively correlated with the cell traction force (Supplementary Fig. 16)^5,47^, these experimental results indicate that VIFs impact cellular forces in a matrix stiffness-dependent manner.

### Disruption of vimentin filaments affects microtubule organizations in a compression-dependent manner

Our results thus far show vimentin plays a key role in tuning cell-generated internal forces. Vimentin promotes cell traction force when cells are on soft substrates but hinders traction stress on stiff substrates. The experimental TFM results corroborate the predictions of the computational model. Specifically, the model predicts that vimentin strengthens actomyosin-based contractile forces on soft substrates but opposes the intense cell contraction generated on stiff substrates through reinforcement of the microtubule network. Consequently, the absence of vimentin would result in increased compression on microtubules on stiff substrates (Supplementary Fig. 17), potentially leading to their buckling, destabilization, and even depolymerization.

**Fig. 4:**
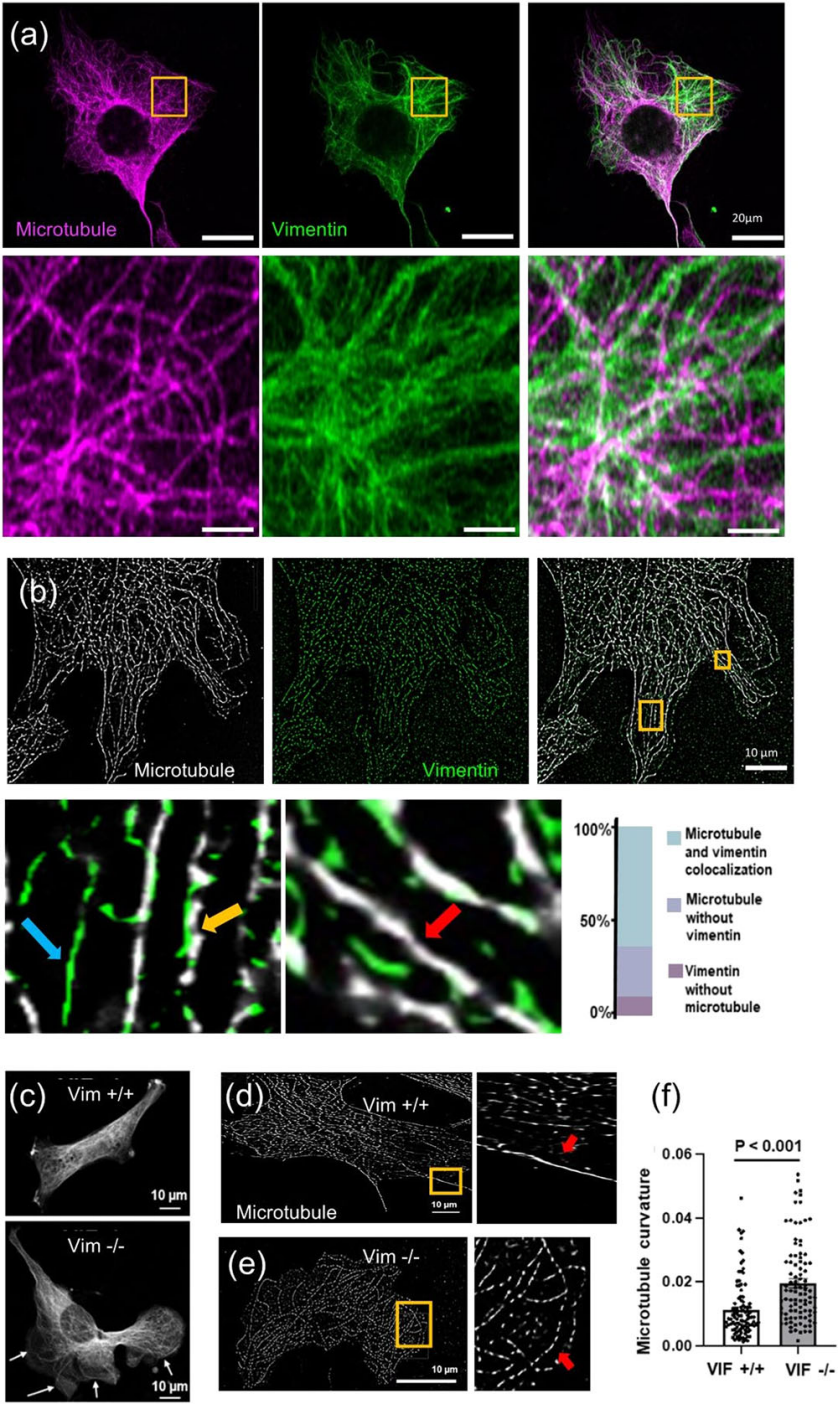
Disruption of vimentin filaments negatively affects microtubule stability causing abnormal buckling of microtubules. **a** Airy-scan images of VIF +/+ cells show that microtubules interpenetrate a dense network of vimentin filaments. **b** STORM images of VIF +/+ cells show that a large fraction of microtubules colocalizes with vimentin filaments. Yellow, red, and blue arrows denote vimentin-microtubule colocalization, microtubule track without vimentin, and vimentin track without microtubule, respectively. **c** Representative confocal images of microtubules in VIF +/+ and VIF −/− cells. Arrows in VIF −/− image highlight long curved microtubule filaments or bundles not seen in VIF +/+ cells. **d** STORM images of microtubules in VIF +/+ and (**e**) VIF −/− cells. Zoomed-in images show differences in microtubule curvature. **f** Microtubule filament curvature analysis from STORM images. Approximately 100 microtubule filaments were analyzed from 10 cells (10 filaments per cell) with 2 independent trials per condition. Two-sided unpaired Student’s t-test was used.

We therefore inferred that there may be detectable differences in microtubule filament structure in cells with and without vimentin on stiff substrates. Thus, we next examine the microtubule organization in VIF +/+ and VIF −/− fibroblasts cultured on rigid substrates (Fig. 4). Airy-scan confocal images of wild-type fibroblasts show that microtubules interpenetrate a dense network of intermediate filaments (Fig. 4a). There is a noticeable extent of colocalization between vimentin and microtubule filaments and the two networks interweave, suggesting vimentin may reinforce and buttress microtubule networks against compressive forces. Note that vimentin filaments physically interact with microtubules^43^, and form connections with microtubules through cytolinker proteins like plectin^19^. However, it should be acknowledged that while these interactions necessitate the colocalization of vimentin and microtubules, such colocalization does not inherently indicate a direct physical interaction between vimentin and microtubules.

To quantitatively measure the vimentin-microtubule colocalization, we next use STORM microscopy which gives single-molecule details and a resolution of 20 nm in the X-Y plane (Fig. 4b). The analysis of our STORM images shows that a large fraction of microtubules colocalizes with vimentin filaments (Fig. 4b). We next study how lack of this mechanical interaction in VIF −/− fibroblasts affects the microtubule organization. We find that, compared with wild-type fibroblasts, microtubules in VIF −/− cells exhibit lower stability (i.e., microtubules in VIF −/− are unable to withstand actomyosin-based compressive forces and maintain their normal shape). This is shown in Fig. 4c (white arrows) where microtubules in VIF −/− cells buckle at much larger wavelengths resulting in abnormal microtubule shapes under contractility-based compressive stresses. To quantitatively measure the change in microtubule organization in the absence of vimentin, we calculate the curvature of the microtubules in STORM images of VIF +/+ and VIF −/− cells. Our results show that microtubules in VIF −/− fibroblasts are unable to withstand actomyosin-based compressive forces and exhibit higher curvatures, indicating that lack of vimentin causes instability of microtubules under compression (Figs. 4d–f).

### The state of mechanical stress in the cytoskeleton determines the spatial distribution of vimentin filaments

**Fig. 5:**
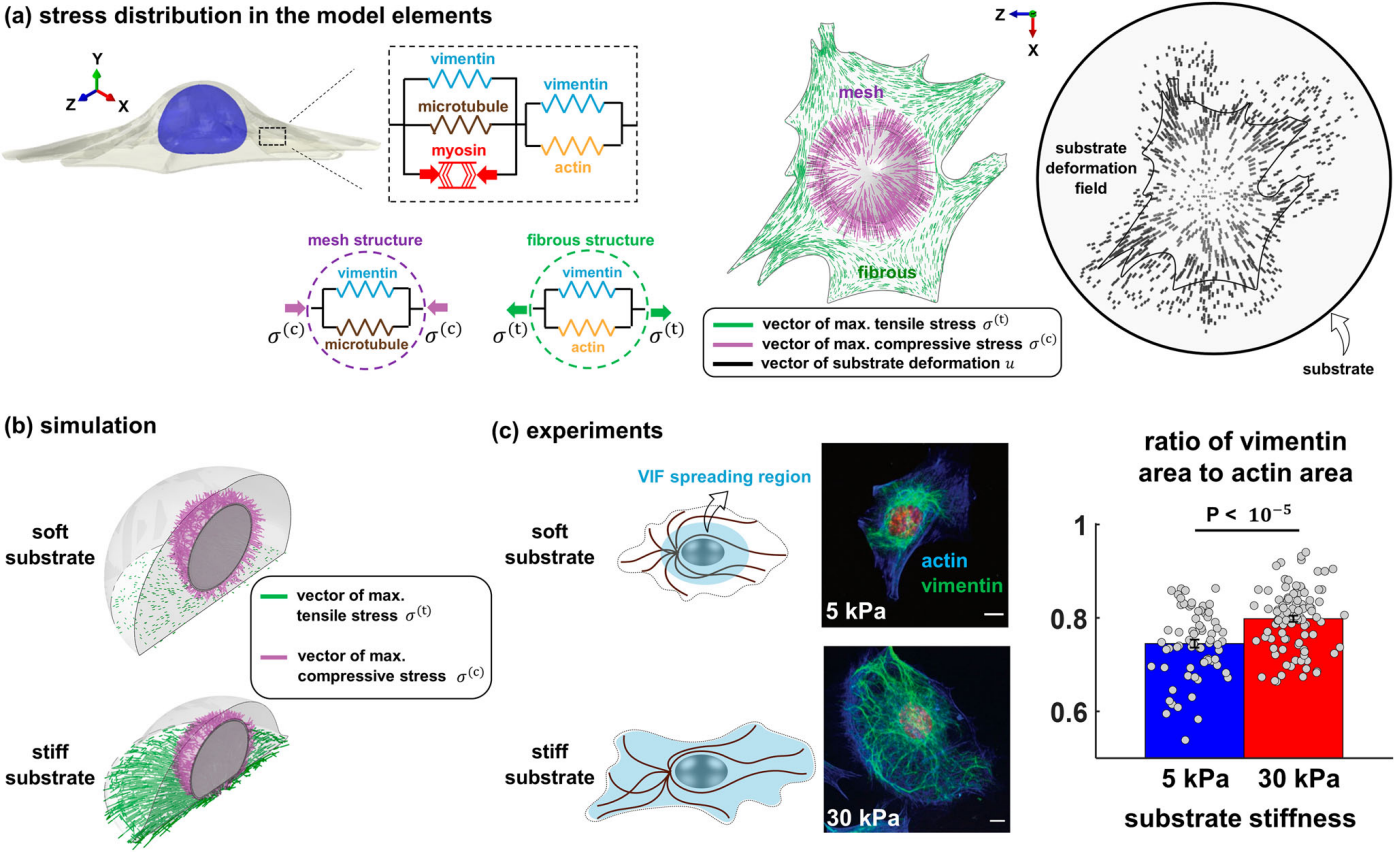
The effect of substrate stiffness on vimentin organization. **a** Using finite element simulations, a fibroblast with a random shape is simulated on a stiff substrate as a 3D continuum of representative volume elements (RVEs), each of which is composed of five elements. As the active element (representing myosin motors) generates internal contractile forces, the elements in parallel with the active element experience compression, while the elements in series undergo tensile stresses. We determine the maximum compressive stress ${\sigma }^{({{{{{\rm{c}}}}}})}$ and the maximum tensile stress ${\sigma }^{({{{{{\rm{t}}}}}})}$ that the compressive and tensile elements experience, respectively, at each RVE. **b** To study how substrate stiffness impacts ${\sigma }^{({{{{{\rm{c}}}}}})}$ and ${\sigma }^{({{{{{\rm{t}}}}}})}$, we simulate cells on soft and stiff circular micropatterned substrates. Our simulations show that, while the tensile stress ${\sigma }^{({{{{{\rm{t}}}}}})}$ is remarkedly disrupted on soft substrates, cells on both substrates experience high compressive stress ${\sigma }^{({{{{{\rm{c}}}}}})}$ around the nucleus in the direction perpendicular to the nuclear envelope, expecting the existence of wavy and compressed VIFs in the juxtanuclear region of cells on both substrates. **c** In agreement with the simulations, our experiments show formation of mesh-like vimentin networks around the nucleus on both soft and stiff substrates. Furthermore, concomitant with the increased tension in the tensile vimentin element predicted by the model, our experiments show that cells on the stiff substrate form vimentin fibers that can reach the cell periphery, indicating that vimentin fibers extend toward the cell periphery with increasing cytoskeletal tension. ($n$ = 70 and 99. The unpaired Student’s t-test was used. The height of the bars and the error bars indicate the mean and the standard error, respectively. Scale bars: 10 μm).

Since the model has two VIF elements interacting with the actin and microtubule networks, we next ask (i) how mechanical stresses are spatially distributed in each of these VIF elements, and (ii) how these mechanical stresses change with matrix stiffness. Note that as the myosin element generates contractile forces, the elements in parallel with the myosin element experience compression, while the elements in series are subject to tensile stresses and promote the transmission of tensile forces to the ECM (Fig. 5a). Our three-dimensional model enables us to measure the stress that each of these elements experiences in different directions. Note that cells can experience different stress levels in different directions and therefore stress should be considered as a direction-dependent quantity. Thus, as detailed in Supplementary Note 1, we first determine the 3D stress tensors for the compressive (parallel) ${{{{{{\rm{\sigma }}}}}}}_{{ij}}^{({{{{{\rm{c}}}}}})}$ and tensile (series) ${{{{{{\rm{\sigma }}}}}}}_{{ij}}^{({{{{{\rm{t}}}}}})}$ elements. We then calculate the minimum eigenvalue (minimum principal value) of the stress tensor ${{{{{{\rm{\sigma }}}}}}}_{{ij}}^{({{{{{\rm{c}}}}}})}$ to determine the maximum compressive stress that the compressive elements experience at any point in the cytoskeleton. Similarly, we calculate the maximum eigenvalue of ${{{{{{\rm{\sigma }}}}}}}_{{ij}}^{({{{{{\rm{t}}}}}})}$ to determine the maximum tensile stress that the tensile elements spatially experience (Fig. 5a). We finally plot the maximum compressive stress and the maximum tensile stress as vectors, ${\sigma }^{({{{{{\rm{c}}}}}})}$ and ${\sigma }^{({{{{{\rm{t}}}}}})}$, whose orientations represent the maximum compression direction (purple lines in Fig. 5a) and the maximum tension direction (green lines in Fig. 5a) in the parallel and series elements, respectively.

#### Model predictions

To study how matrix stiffness impacts ${\sigma }^{({{{{{\rm{c}}}}}})}$ and ${\sigma }^{({{{{{\rm{t}}}}}})}$, we simulate cells on soft and stiff circular micropatterned substrates (Fig. 5b). As expected, the model predicts that the maximum tensile stress ${\sigma }^{({{{{{\rm{t}}}}}})}$ decreases in cells on the soft substrate (green line in Fig. 5b), and therefore VIFs that interact with actomyosin filaments and undergo tensile stresses are expected to experience lower tension on soft matrices, particularly around the cell periphery. However, in contrast to ${\sigma }^{({{{{{\rm{t}}}}}})}$, we find that the maximum compressive stress ${\sigma }^{({{{{{\rm{c}}}}}})}$ remains high in the juxtanuclear region with softening of the substrate (purple line in Fig. 5b). This indicates that VIFs that undergo compressive stresses experience high levels of compression in the juxtanuclear region on both soft and stiff matrices, and therefore vimentin filaments are expected to appear more wavy, compressed, and buckled around the nucleus on both substrates.

#### Experiments

To test whether the matrix stiffness-induced changes in the stress field affect vimentin organization, we culture cells on substrates with different stiffness (Fig. 5c) and we study how the spatial distribution of vimentin changes with matrix stiffness. In agreement with the model prediction, our experiments show the appearance of wavy mesh-like VIFs in the juxtanuclear region^48^, which form a cage around the nucleus on both soft and stiff substrates (Fig. 5c, Supplementary Fig. 18). Interestingly, concomitant with the increased tension in tensile VIFs predicted by the model, our experiments show that cells on the stiff substrate form vimentin fibers that can reach the cell periphery (Fig. 5c). Together, our results show that vimentin filaments extend toward the cell periphery with increasing ECM stiffness.

### Vimentin intermediate filaments contribute to the long-range propagation of local forces in the cytoplasm

**Fig. 6:**
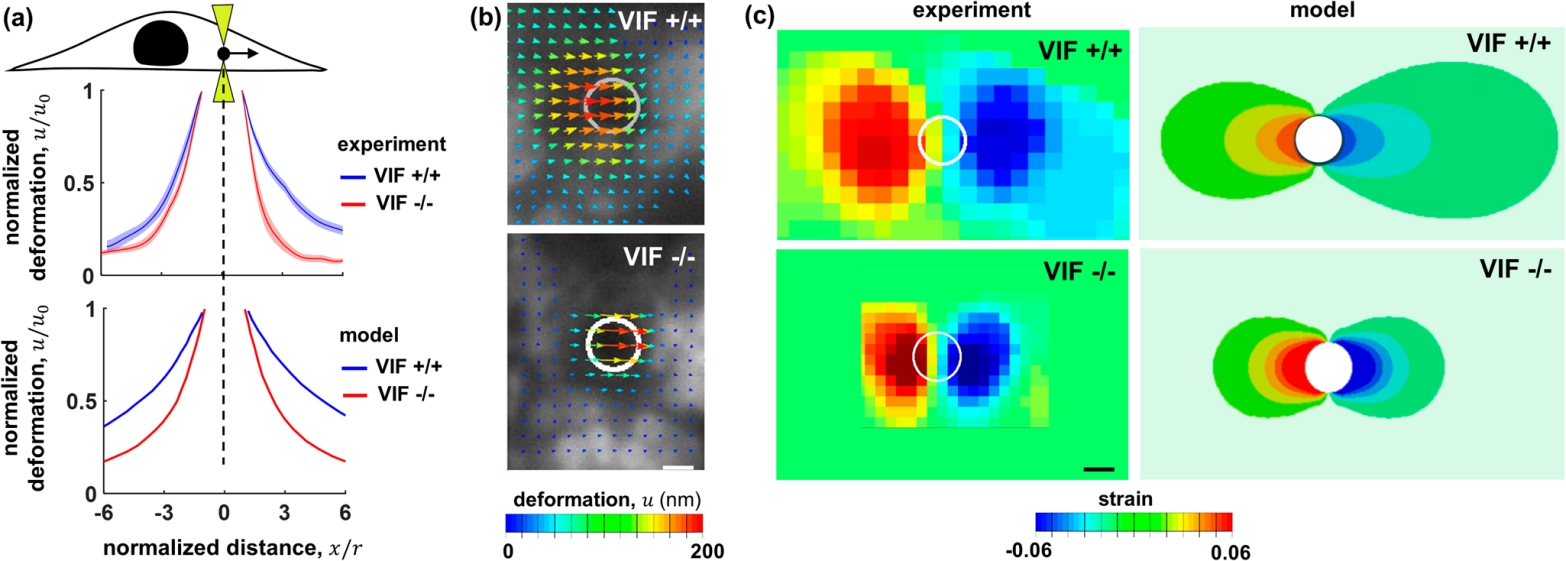
The effect of vimentin on the propagation of local forces in the cytoplasm. The involvement of vimentin in the propagation of both tensile and compressive forces is further illustrated in optical tweezer experiments where a bead with a radius of $r$ = 1 μm is dragged in the x-direction in the cytoplasm over ${u}_{0}$ = 200 nm. Visualizing the movement of surrounding fluorescently-labeled mitochondria shows the effect of vimentin in the transmission of local forces in the cytoplasm. **a** The displacement field along the x-direction in the cytoplasm is plotted as a function of the distance from the bead for VIF −/− and VIF +/+ cells. The solid lines and the semitransparent areas around the solid lines represent the mean and standard error, respectively, with at least $n$ = 10 for each curve. **b** A representative image of the displacement field in the cytoplasm around the bead (white circle). **c** A representative image of the strain field around the bead (white circle) determined as the derivative of the displacement field. As the bead moves in the cytoplasm in the x-direction, compressive (negative strain) and tensile (positive strain) fields are generated in the front and back of the bead, respectively, which extend farther in wild-type fibroblasts than in VIF −/− cells. Scale bars: 1 μm.

To further study the role of vimentin in cellular force transmission, we compare the model to optical tweezer microscopy experiments where we generate a local force field in the cytoplasm and measure how the generated force propagates in the cytoplasm with and without the presence of vimentin filaments^49^. In our optical tweezer microscopy experiments, a 1 μm radius ($r$) bead is dragged in the cytoplasm over 200 nm (${u}_{0}$) while the resulting displacement and strain fields generated around the bead in the cytoplasm are measured by visualizing the movement of surrounding fluorescently labeled mitochondria (Fig. 6a, b). It should be noted that the movement of mitochondria was used here as an indirect indicator of cytoplasm displacement, and that cytoplasm displacement was not measured directly. As the bead moves in the cytoplasm, compressive (negative strain) and tensile (positive strain) fields are generated in the front and back of the bead, respectively (Fig. 6c).

#### Model predictions

Intermediate filaments are known to exhibit nonlinear strain-stiffening behavior^15–18,50^, and the model predicts that the strain-stiffening property of vimentin filaments can lead to long-range propagations of the displacement field generated by the bead movement. This is better shown in Fig. 6a where we plot the local cytoplasmic displacement along the drag direction, $u$, as a function of the distance to the bead, $x$. As vimentin filaments undergo compressive and tensile deformations in the front and back of the moving bead, respectively, the strain-stiffening property of vimentin filaments enables them to exhibit higher resistance against deformation (Supplementary Fig. 19) which in turn leads to long-range propagation and slow decay of the deformation and strain fields (Fig. 6a–c).

#### Experiments

To test the role of vimentin filaments in the propagation of local forces, we used our optical tweezer microscopy method for VIF +/+ and VIF −/− cells where the bead was dragged at a speed of 2 µm/s (original data from our previous published data in ref. ^49^). Our experimental results showed that both compressive and tensile fields extended farther in wild-type fibroblasts than in VIF −/− cells. Furthermore, our results showed that vimentin also protects the other cytoskeletal components under loading, as lack of vimentin in VIF −/− cells caused cytoskeletal damage in optical tweezer experiments which in turn led to strain-softening and fast decay of the displacement field in VIF −/− cells. Together, our results show that vimentin filaments not only are involved in the propagation of local forces in the cytoskeleton but also increase the range of force propagation.

## Discussion and conclusions

Our results elucidate that vimentin can exhibit matrix stiffness-dependent effects on traction forces as vimentin, directly and indirectly, interacts with other cytoskeletal components (i.e., the actomyosin and microtubule networks) whose state and organization change with matrix stiffness. We show that VIF −/− cells exhibit higher spreading area and traction forces on stiff substrates, while on soft substrates they spread less and generate lower traction forces compared with VIF +/+ cells, reconciling prior literature results and in agreement with the predictions of our theory. On the one hand, vimentin is involved in the transmission of actomyosin-based tensile forces to the matrix and therefore enhances traction forces. On the other hand, vimentin reinforces microtubules and their stability under compression, thus promoting the role of microtubules in suppressing cellular traction forces. We show that the competition between these two opposing effects of vimentin is regulated by the microenvironment stiffness.

For high matrix stiffness, the microtubule-reinforcing role of vimentin dominates over its force-transmitting role. This is because cells on stiff matrices develop a strong contractile actomyosin network to transmit tensile forces to the extracellular environment and therefore depletion of vimentin does not markedly decrease the force transmission ability of cells, while microtubules experience high compression, and they require reinforcement from the vimentin network to withstand the compression (Supplementary Fig. 20b). This was shown here via STORM imaging where lack of vimentin in VIF −/− cells changed the buckling modes of microtubules. As a result, cells on stiff matrices experience less resistance against contraction and thus generate higher traction forces, as predicted by the model and supported by our experiments. These results are in agreement with experimental studies in the literature performed on stiff matrices where vimentin-null cells generate higher contractile forces compared with wild-type cells^20,21^.

In contrast, for low matrix stiffness, cells exhibit a weak actomyosin network and thus the force-transmitting role of vimentin becomes more important (Supplementary Fig. 20a). As a result, vimentin increases traction forces in cells on soft matrices, as predicted by the model and supported by our experiments. These results are consistent with traction force microscopy experiments in the literature performed on soft matrices (2.4 kPa) where vimentin-null cells generate lower contractile forces compared with wild-type cells^23,24^.

Our results are also consistent with traction force microscopy experiments of plectin −/− fibroblasts. Plectin is a universal cytolinker protein that interlinks intermediate filaments and anchors them to the nuclear envelope and other organelles in the cytoplasm^51^, forming an interconnected network of biopolymers^19^. Therefore, depletion of plectin in plectin −/− fibroblasts causes aberrant changes in vimentin organization such as loosening of the vimentin network^51^. Vimentin was also found to be less stable and thus more soluble in plectin −/− fibroblasts^52,53^. Importantly, plectin physically links intermediate filaments to actin filaments, myosin filaments, microtubules, and focal adhesions^19^, which all play important roles in the generation and transmission of cellular forces to the extracellular matrix. Thus, depletion of plectin in plectin −/− cells is expected to affect cell traction forces, consistent with traction force microscopic experiments in the literature^54–56^. In agreement with model prediction and similar to the case of VIF −/− cells on soft substrates, plectin −/− fibroblasts generate lower contractile forces on soft substrates of 4 and 8 kPa compared with wild-type cells^57^. In contrast, on rigid substrates, plectin −/− fibroblasts exhibit augmentation of actin stress fibers and focal adhesions compared with wild-type fibroblast cells^58^. All these results show that VIFs interact with the other cytoskeletal components in a matrix stiffness-dependent manner. As a result, disruption of this interaction, by depletion of either vimentin or plectin, can have major matrix-dependent effects on cellular forces.

The theoretical model accounts for myosin motors, actin filaments, microtubules, intermediate filaments, focal adhesions, and the nucleus which are all involved in the generation and transmission of cellular forces to the ECM (Methods). However, it is acknowledged that certain important cellular components, such as integrins and additional actin and microtubule-binding proteins^59–61^, are not explicitly accounted for in our coarse-grained model.

Myosin is represented in our model by an active force-generating contractile element that is connected to the actin element in series, thereby generating tension in the actin element. Modeling myosin and actin as two separate elements can be non-intuitive as these two proteins are not separate entities in cells, but rather form a complex network of actomyosin. The model can be simply reformulated to combine the myosin and actin elements. However, having actin as a separate element enables us to better illustrate that in a contractile cell actin filaments and microtubules experience tensile and compressive forces, respectively, and thus vimentin can undergo both tension and compression as it mechanically interacts with actin and microtubules. Also, note that myosin contractile forces can also generate compression in actin filaments as observed in in vitro experimental models of actomyosin networks^62^. However, the compressive stresses are relieved through buckling and severing of actin filaments, keeping only tensile forces in the actin network^62^. Therefore, we assume that the actin element only experiences tension in the model and transmits the tensile forces to the matrix through focal adhesions as observed experimentally^35,63,64^. Also, note that actin filaments form diverse contractile structures, including dorsal stress fibers, transverse arcs, ventral stress fibers, the perinuclear actin cap, and cortical actin networks^65^. In our model, the actin element specifically applies to contractile actin structures that connect with the extracellular matrix through focal adhesions, thereby facilitating the transmission of contractile forces to the matrix (e.g., ventral stress fibers).

The cell model responds to matrix stiffening by increasing myosin-generated contractile forces through a feedback mechanism between contractility and cytoskeletal tension (Supplementary Fig. 1). This agrees with experimental observations where the level of phosphorylated myosin motors in fibroblasts increases with matrix stiffness^66^. Concomitant with phosphorylation of more myosin motors and higher cell contractility, the actin element in the model stiffens in the direction of the tensile stresses, representing the recruitment and alignment of actin filaments in response to matrix stiffening^5,6,34,36^. Note that the formation of actin filaments in our simulations colocalizes with phosphorylation of myosin motors as observed experimentally^33^. Starting with isotropic and uniform myosin and actin distributions, Supplementary Fig. 5 shows higher contractility and actin formation in basal regions and close to the cell boundary which is consistent with experimental observations^33,67,68^. Disruption of the actomyosin network in the model, by inhibition of either myosin phosphorylation or actin formation, reduces cellular contractile forces as reported in experimental studies^69^.

Microtubules in the model experience compression as the microtubule element is placed in parallel with the contractile myosin element. Although in vitro models show a complex interaction between the microtubules and actomyosin networks^70^, the intrinsic cell contractility is known to generate compression in a large portion of microtubules which can in turn cause them to buckle^12,14^. Visualization of microtubule dynamics in cells transfected with GFP-tubulin shows that buckling of microtubules increases when cell contractility is stimulated by addition of thrombin to cells, while microtubules buckle less or even completely straighten with decreasing contractility after addition of cytochalasin D to destabilize the actomyosin network^11^. The placement of the microtubules in parallel with the active element in the model is consistent with these experimental observations.

The model predicts that cell contractility, traction force, and matrix deformation increase with disruption of microtubules, which are all consistent with experimental studies^39^. Kolodney and Elson showed that depolymerization of microtubules upon nocodazole treatment increases cell contractility by promoting phosphorylation of myosin light chains^9^. The increase in cell contractility was found to be associated with stimulation of actin and focal adhesion organizations and formation of stress fibers^71^. It was later shown that GEF-H1 is involved in this nocodazole-induced increase in contractility. GEF-H1 is a RhoA-specific guanine nucleotide exchange factor which is activated by microtubule depolymerization^13^. The activated GEF-H1 activates the Rho-Rock pathway, which in turn increases phosphorylation of myosin motors and cell contractility. Consistent with the increase in cell contractility, other studies showed that depolymerization of microtubules also leads to higher cell force generation in fibroblasts^10,72^ which generates larger strains in the underlying substrate^73^.

The model contains two vimentin elements. The first element interacts with microtubules and stabilizes them under contractility-based compressive forces. Consistently, in vitro studies of isolated microtubules showed that microtubules without the VIF reinforcement are less stable, buckle at much larger wavelengths, and withstand remarkedly smaller compressive stresses compared with VIF-reinforced microtubules in living cells^14^. Other experimental studies also showed that VIFs template and stabilize microtubule organizations as vimentin turns over much slower than microtubules^42^. Furthermore, recent evidence from in vitro studies shows that VIFs stabilize microtubules against depolymerization through direct physical interactions^43^. All these studies indicate that vimentin filaments support and stabilize microtubules under contractility-based compressive stresses and prevent them from destabilization.

We discussed that in cells with high levels of actomyosin contractility, microtubules experience high actomyosin-based compression in the absence of vimentin filaments leading to instability (Fig. 4), reorganization (Supplementary Fig. 17d), and even depolymerization (Supplementary Fig. 17b) of microtubules. This agrees with recent studies on fibroblasts where disruption of actomyosin-based compressive forces on microtubules allows them to grow in length and number^74^. In addition to fibroblasts, the same behavior has been recently reported in glioblastoma cells where disruption of actomyosin contractility, and subsequently compressive forces on microtubules, increase the length and number of microtubules^75^. Our results are also supported by studies of isolated microtubules where compression on microtubules reduces the rate of microtubule growth^76,77^ and increases the occurrence of microtubule catastrophe^78^. Furthermore, depletion of VIFs on stiff substrates has been reported to increase (i) the dynamics of GEF-H1, (ii) the level of active GEF-H1, (iii) phosphorylation of GEF-H1 on Ser886, (iv) guanine nucleotide exchange activity of GEF-H1 towards RhoA, and (v) subsequently the expression level of tropomyosin 4 which plays a key role cell contraction^20^. Note that depolymerization of microtubules is known to activate GEF-H1^13^ which in turn activates the Rho-Rock pathway to increase phosphorylation of myosin motors and cell contractility^9^. This may indicate that the increase in contractility upon VIF depletion can be due to depolymerization of microtubules. Note that activation of GEF-H1 in VIF −/− cells is not directly measured in this study. However, our TFM experiments show that VIF −/− cells generate higher traction forces on stiff substrates compared to VIF +/+ cells, whether due to microtubule depolymerization and subsequently GEF-H1 activation or due to the diminished resistance of microtubules against compression, resulting in reduced resistance against cell contraction. Also, note that immunoblot analyses of cell lysates from VIF +/+ and VIF −/− fibroblasts did not show a remarkable difference in microtubule expression levels^79^. This indicates that the total amount of microtubules (both polymerized and depolymerized) measured by immunoblotting remains the same and the lack of vimentin in VIF −/− fibroblasts only affects microtubule stability, organization, and depolymerization rate as observed in Fig. 4, Supplementary Fig. 17d, and Supplementary Fig. 17b, respectively.

In addition to reinforcing microtubules under compression, vimentin is also known to interact with actin filaments. This interaction is captured by the second vimentin element in the model, and we show that the vimentin-actin interaction becomes more important in cells with low actomyosin levels (e.g., cells on soft substrates) as depletion of vimentin in these cells decreases contractile forces. This agrees with recent experimental studies showing that vimentin and actin filaments form an interpenetrating network, and lack of vimentin in VIF −/− fibroblasts on soft substrates reduces traction forces^24^. This vimentin element in our model experiences tension which is consistent with experimental observations. Recently, in situ nonlinear Raman imaging of cells on 2D rigid substrates showed that vimentin filaments can experience tensile stresses due to the intrinsic contractility-driven cytoskeletal tension^44^ which can, in turn, lead to unfolding of coiled-coil α-helical structures in vimentin filaments into anti-parallel β-strand structures^80^. Furthermore, the tension-driven unfolding of vimentin filaments was found to decrease with disruption of cytoskeletal tension upon culturing cells on soft substrates or using actomyosin inhibitors including blebbistatin and latrunculin A^44^. Similarly, vimentin filaments in “tensegrity models” are assumed to undergo tensile stresses^11^. Also, “actomyosin-associated vimentin intermediate filaments” have been shown to play a critical role in the transmission of tensile stresses between the nucleus and the ECM^81^. All these studies show that vimentin filaments are involved in the transmission of contractility-based tensile forces to the ECM through interactions with contractile actin filaments, and our results show that the balance between vimentin-actin and vimentin-microtubule interactions regulates the effect of vimentin on cellular forces.

It should be noted that individual actin filaments undergo minimal stretching before reaching a breaking point when subjected to tension^82,83^. Therefore, while the addition of the vimentin element in our model enhances the overall stiffness of the elements in series, subsequently reinforcing the actin element and reducing its stretching under tension, it is important to clarify that this augmentation does not imply a direct stiffening of individual actin filaments or prevention of their stretching. Instead, this enhancement indicates that in the presence of “actomyosin-associated vimentin intermediate filaments”^81^, there is an augmentation in the integrity and stiffness of the entire network of interconnected actomyosin filaments, which facilitates the transmission of forces, as evidenced by our optical tweezer experiments.

Our results showed that with increased tension in the cytoskeleton due to increasing substrate stiffness, fibrous vimentin filaments extend toward the cell periphery, a phenomenon not observed in cells on soft substrates (Fig. 5c). However, it should be noted that cells on stiff substrates exhibit greater spreading. Therefore, to test whether increased cytoskeletal tension alone is sufficient to induce the extension of vimentin filaments towards the cell periphery, cells should be cultured on micropatterned substrates with varying substrate stiffness. This approach allows for the measurement of vimentin filament extension towards the cell periphery as a function of substrate stiffness while maintaining a constant cell spreading area.

Taken together, our study elucidates the complex crosstalk between vimentin, actomyosin, and microtubules which impacts cell-generated traction forces in a matrix stiffness-dependent manner. Vimentin is involved in various important biological processes including migration^84^, polarity^85^, EMTs^86^, cataracts^87^, and cancer progression^88^. Given that cellular traction forces are central to both wound healing and a wide range of pathological processes including fibrosis and surgical adhesions, our study has broad implications for understanding the effect of vimentin on cell-generated traction forces within different physiological and pathological microenvironments.

## Methods

### Cell culture, reagents, immunostaining

Mouse embryonic fibroblasts were derived from wild-type and vimentin null mice and immortalized by stable expression of SV40 large T-antigen (kindly provided by J. Ericsson, Abo Akademi University, Turku, Finland)^25^. Cells were grown in 1X DMEM (Life Technologies; Catalog no: MT10013CV) supplemented with 10% fetal bovine serum (GE Healthcare Life Sciences, Catalog no: SH3008803), 1% penicillin-streptomycin (Gibco) and nonessential amino acid (Life Technologies), and 10 mM HEPES and sodium pyruvate (Life Technologies) at 37 °C with 5% CO_2_. Cells were plated at a density of 10,000 cells/gel or less.

For immunofluorescence experiments, cells were fixed with 4% paraformaldehyde (Affymetrix) followed by 5% BSA and 1% Saponin (Sigma) for blocking and permeabilization. Primary antibodies were Alexa- Fluor 647 phalloidin (Invitrogen Catalog no: A22287), anti-vimentin (Novus Biologicals Catalog no: NB300-223), and dapi (Sigma Catalog no: D9542). Alpha-tubulin rat antibody (Bio-Rad- Catalog no: MCA77G) at a concentration of 1:200 was used for detecting microtubules.

### Western blot analysis

Wild-type and vimentin-null mouse embryonic fibroblast cells were cultured in a 25 cm^2^ culture flask until approximately 90% confluence. Mouse primary hepatocytes were generously provided by the Wells Lab at the University of Pennsylvania. Cells were lysed in the presence of protease inhibitor cocktail and the concentrations of whole cell proteins were determined by protein BCA assay (Thermo Fisher Scientific). Equal amount of whole cell proteins was subjected to Western blot analysis with the following primary antibodies: mouse anti-β-actin (Abcam), rabbit anti-vimentin (Cell Signaling Technology), and mouse anti-cytokeratin pan (Sigma), and the following secondary antibodies: 680RD donkey anti-rabbit, 680RD donkey anti-mouse, and 800CW donkey anti-mouse (Licor). Targeted protein bands were imaged with an Odyssey Infrared Imaging System (Supplementary Fig. 22).

### Microscopy and Imaging

Images of the cell and beads are acquired with a Leica DMIRE2 microscope or Leica DMi8 equipped with a spinning disk confocal unit using iVision software. An environmental chamber is used to maintain the temperature at 37 °C and 5% CO_2_ for live-cell imaging. Bright-field images of cells and fluorescent images of the beads are acquired at multiple positions with a 40X objective.

### Cell traction force microscopy

To perform traction force microscopy experiments, polyacrylamide hydrogel substrates of desired stiffness with 1% of 200 nm fluorescently labeled green beads (2% solid, Thermo Fisher Scientific) were prepared as described in ref. ^89^. The concentration of acrylamide and bisacrylamide varied for different matrix stiffness. Substrates were coated with 50 µg/ml of either collagen type I (Corning) or fish fibronectin (homemade); Fibronectin was purified from the blood plasma of farmed salmon (Sea Run Holding, Eastport ME) by affinity chromatography using gelatin-agarose beads^90^ and eluted by 1 M arginine^91^, followed by dialysis in PBS. Cell boundaries were determined manually using the phase contrast images of the cell. After 24 h of plating cells, phase images of the cell, stressed and relaxed images of fluorescently labeled particles were acquired using an epifluorescence microscope with an environmental chamber, which allowed us to determine the displacement field using particle image velocimetry. Image acquisition was performed as we maintained 37 °C and 5% CO_2_ throughout the experiments. For the traction force microscopy analysis, a custom-built Matlab code was used. From the displacement field, we calculated the root mean square (RMS) values of cellular contractile forces per unit area using constrained Fourier Transform Traction Microscopy^92^. No regularization was used in the traction calculation. The details of the calculation can be found in^93^.

### STORM Imaging

STORM images of microtubules (α-tubulin) and vimentin filaments of wild-type and vimentin-null cells were obtained using ONI Nanoimager equipped with 100X oil immersion objective with 1.5 NA in TIRF mode and s-CMOS camera. The images were processed using NimOS software. The kappa-curvature analysis plugin from FIJI-ImageJ was used to quantify the curvature of the filaments.

### Microtubule filament curvature analysis

Approximately 100 microtubule filaments were analyzed from 10 cells (10 filaments per cell) from 2 independent trials per condition. The filaments were selected randomly independent of whether they overlapped with vimentin or not. Only those filaments were selected which could be traced from the periphery towards the inner region of the cell. Minimum length traced was around 90 µm. Only single cells were taken into account. Any bi-nucleated cells were excluded.

### Cell area determination

The cell area covered by vimentin and actin filaments was determined from optical images collected with a 40x objective. Cells were imaged for both actin and vimentin. The cell area was measured using ImageJ software by manually training the periphery of single cells from the actin image channel. Next, the area of the vimentin cytoskeletal filaments was manually traced. For individual cells, the ratio of vimentin area to actin area was then computed. Only single cells were taken into account. Any bi-nucleated cells were excluded.

### Theoretical model

To study the role of vimentin in cell responses to matrix stiffness, we developed a theoretical model for three-dimensional and one-dimensional frameworks (see Supplementary Note for more details and Supplementary Table 1 for the model parameters used in our simulations). All of the relevant features of the three-dimensional framework are present in our one-dimensional framework, which also serves to highlight the main concepts underlying our chemo-mechanical formulation. The cell model in the one-dimensional framework is composed of the myosin motors, the microtubules, the actin filaments, and the vimentin filaments, and is connected to a linear matrix model as shown in Supplementary Fig. 21 (see Supplementary Note 2). Supplementary Equations S2.1-7 demonstrate the constitutive behavior of all components of the model and their interactions. The cell model in the three-dimensional framework includes the following components: the cytoskeleton, the focal adhesions, and the nucleus. We used the finite element method (FEM) to solve the equations of the three-dimensional framework and to perform simulations. In FEM, equations are solved by discretizing a physical domain into a continuum of finite-sized representative volume elements (RVEs), which are interconnected through element nodes. The equations are approximated over each RVE, and the resulting system of equations is solved to obtain the solution over the entire domain. The equations for the cytoskeleton model are fully described in Supplementary Note 1, and similar to the one-dimensional framework, connect the cytoskeletal components (the myosin motors, the microtubules, the actin filaments, and the vimentin filaments) as shown in Fig. 1a. All FEM simulations were performed with an initially uniform (independent of spatial location) and isotropic (independent of direction) condition; e.g., the initial contractility of the myosin element and the initial stiffness of the actin, microtubule, and vimentin elements were the same in all RVEs (uniform), and in each RVE they had the same value in all directions (isotropic). Starting with the uniform and isotropic conditions, our three-dimensional simulations predict the nonuniform and anisotropic reorganizations of each of the cytoskeletal components (Fig. 5, Supplementary Figs. 5, 16, 17) and how their reorganizations contribute to the transmission of cellular forces to the extracellular environment. Focal adhesions in the three-dimensional framework are treated as a set of initially soft nonlinear mechanical elements that stiffen with tension to capture the tension-dependent formation of the focal adhesions. When the tensile stress exerted by the contractile cell to the adhesion layer exceeds a certain threshold, mature focal adhesions are formed, and the cell is connected to the substrate, while below this threshold, the stiffness of the adhesion layer remains low and the substrate experiences negligible forces. The nucleus is modeled as a fibrous elastic thin layer (representing the nuclear envelope and lamina layer) filled with a solid material which represents chromatin and other subnuclear components (see^33^ for more details). The matrix is modeled as a thick linear elastic material. The theoretical model focuses on the stationary behavior of fibroblasts where cells have fully spread on the substrate. Note that the model can be readily extended to study the time-dependent behavior of cells as described in our recent publications^94^. Similarly, our experiments are performed in the stationary configuration where cells have fully spread on the substrate. Also, to ensure the accuracy of the three-dimensional simulations, mesh convergence studies, as are typically performed in finite element simulations, were conducted where the simulations were repeated with successively finer mesh sizes until the simulations results converged to a certain level of accuracy and further mesh refinement did not change the results.

### Optical tweezer microscopy

1-μm radius carboxylate-modified polystyrene spherical beads (from Molecular Probes) were delivered into mouse embryonic fibroblast cells through endocytosis. Before the experiment, the beads were incubated with the cells for 12 h to ensure that they were thoroughly endocytosed. To avoid disrupting cell behavior, the number of beads was limited to less than 5 per cell. To minimize the effect of interactions with the nuclear envelope and the cell membrane in our mechanical measurements, we used only beads that were at least 1.5 μm deep into the cell and away from the nucleus. The details of the measurement of intracellular displacement and strain fields upon the bead movement in the cytoplasm can be found in our previous work^49^. Briefly, we first used a charge-coupled device (CCD) camera (ORCA ER Hamamatsu) to take images of fluorescently labeled mitochondria (Mito Tracker Green FM, Invitrogen) and the bead at a rate of 30 frames per second at ×100 magnification. Using particle image velocimetry analysis (PIVlab in MATLAB) of fluorescently labeled mitochondria, we then determined the cytoplasmic displacement field where 64 × 64 pixels segments from the reference image (taken before the bead movement) were cross-correlated with corresponding 64 × 64 pixels segments of the target image (during bead movement). The position of the highest cross-correlation was taken as the displacement of the image segment. The strain field was calculated as the first derivative of the displacement field as shown in ref. ^49^. For more details on optical tweezed microscopy, please refer to our previous publication^49^.

### Statistics and reproducibility

The statistical analyses conducted on the data and the sample sizes in each figure were described in their respective figure captions. Experiments were performed with at least two independent repeats. The original data can be found in the Supplementary Data file.

### Reporting summary

Further information on research design is available in the Nature Portfolio Reporting Summary linked to this article.

### Supplementary information

Supplementary Information

Description of Additional Supplementary Files

Supplementary Data

Reporting Summary

## Data Availability

The source data behind the graphs in the paper can be found in Supplementary Data. Any remaining data that support the findings of this study are available from the corresponding author upon reasonable request. Uncropped/unedited blot images are presented in Supplementary Fig. 22.

## Code availability

Custom code used in this study is available from the GitHub repository https://github.com/Farid-Alisafaei/Vimentin_Mechanobiology_TFM^95^.
